# Prdx6 Upregulation by Curcumin Attenuates Ischemic Oxidative Damage via SP1 in Rats after Stroke

**DOI:** 10.1155/2017/6597401

**Published:** 2017-05-17

**Authors:** Gongwei Jia, Botao Tan, Jingxi Ma, Lina Zhang, Xinhao Jin, Changqing Li

**Affiliations:** ^1^Department of Rehabilitation, The Second Affiliated Hospital of Chongqing Medical University, 76 Linjiang Road, Yuzhong District, Chongqing 400010, China; ^2^Department of Neurology, The Second Affiliated Hospital of Chongqing Medical University, 76 Linjiang Road, Yuzhong District, Chongqing 400010, China

## Abstract

**Background:**

The role of Peroxiredoxin 6 (Prdx6) in brain ischemia remains unclear. Curcumin (Cur) treatment elicits neuroprotective effects against cerebral ischemic injury, and the associated mechanisms may involve Prdx6. In this study, we investigated whether Prdx6 and the transcription factor specific protein 1 (SP1) were involved in the antioxidant effect of Cur after stoke.

**Methods:**

Focal cerebral ischemic injury was induced by transient middle cerebral artery occlusion for 2 hours in male Sprague-Dawley rats treated with or without Prdx6 siRNA. Expression of Prdx6 in the penumbra was assessed by Real-Time PCR (RT-PCR), Western blot analysis, and immunoflourescent staining. In addition, infarct volume, neurological deficit score, and oxidative stress were evaluated. Prdx6 levels were also determined in the presence and absence of SP1 antagonist mithramycin A (MTM-A).

**Results:**

Cur treatment upregulated Prdx6 protein expression and the number of Prdx6-positive neuronal cells 24 hours after reperfusion. Cur treatment also attenuated oxidative stress and induced neuroprotective effects against ischemic damage, whereas the beneficial effects of Cur treatment were lost in animals treated with Prdx6-siRNA. Prdx6 upregulation by Cur treatment was abolished by SP1 antagonists MTM.

**Conclusions:**

Prdx6 upregulation by Cur treatment attenuates ischemic oxidative damage through SP1 induction in rats after stroke. This represents a novel mechanism of Cur-induced neuroprotection against cerebral ischemia.

## 1. Introduction

Ischemic stroke is one of the leading causes of morbidity and mortality in China [1]. In the treatment of ischemic stroke it is vital that reperfusion occur as quickly as possible to help alleviate cerebral ischemic injury. However, a phenomenon known as cerebral ischemia/reperfusion (I/R) injury can arise, which can also cause brain injury again. The mechanism of cerebral I/R injury is a complex cascade of pathophysiological events including oxidative stress, apoptosis, inflammation, and excitotoxicity, although the mechanisms are not fully elucidated [2]. Oxidative stress may be an initiating factor [3], although oxidative stress, apoptosis, and inflammation are observed in parallel in the pathogenesis of ischemic stroke. Oxidative stress is caused by the imbalance between production of free radicals and antioxidant defenses [4].

Peroxiredoxin 6 (Prdx6) is the sixth mammalian peroxiredoxin isoform and has a single conserved cysteine residue [5]. Prdx6 is a bifunctional protein with glutathione peroxidase and calcium-independent phospholipase A2 (PLA2) activity [6]. Prdx6 knockout mice are more susceptible to ischemic reperfusion injury in the heart, as evidenced by increased numbers of apoptotic cardiomyocytes [7]. Additionally, Prdx6 knockout mice are more susceptible to liver ischemic reperfusion injury with increased mitochondrial dysfunction and generation of H_2_O_2_ [8]. In vitro, Prdx6 can alleviate damage to neuronal cells by regulating ROS levels [9]. 4-Hydroxybenzyl alcohol (4-HBA), a herb-derived phenolic compound, has therapeutic effects on cerebral ischemic reperfusion injury by increasing Prdx6 in vivo and in vitro [10].

Curcumin (Cur) is a phenolic compound that is extracted from* Curcuma longa* that displays neuroprotective effects as an antioxidant, antiapoptotic, and anti-inflammatory molecule in cerebral ischemic reperfusion [11, 12]. A recent study verified that Cur upregulated Prdx6 expression and attenuated ROS-based endoplasmic reticulum (ER) stress in mouse hippocampal cells [13]. In human lens epithelial cells, Cur reduced ROS-mediated apoptosis by upregulation of Prdx6 in a manner dependent on the activity of the transcription factor specificity protein 1 (SP1) [14]. Interestingly, SP1 is expressed in HT22 cells and stroke-induced mice [15].

Based on these findings, we investigated whether Prdx6 is involved in the neuroprotective effects of Cur treatment through SP1. Our hypothesis is that Cur exerts neuroprotective effects through activation of Prdx6/SP1 during a stroke.

## 2. Materials and Methods

### 2.1. Animals and Drugs

Adult male Sprague-Dawley rats weighing 250–300 g were purchased from Chongqing Medical University. Rats were kept under controlled conditions (25 ± 1°C, 60–65% humidity, and 12/12 h light/dark cycle) for the duration of the study. Water and food were available ad libitum. All experiments were carried out in accordance with the National Institute of Health guideline requirements in China.

Cur, Mithramycin (MTM), and 2,3,5-triphenyltetrazolium chloride (TTC) were purchased from Sigma (St. Louis, USA). Cur (300 mg/kg) was dissolved in normal saline with 1% dimethylsulfoxide (DMSO) and injected intraperitoneal (IP) 1 hour after middle cerebral artery occlusion following established protocols [16]. MTM (250 *μ*g/kg) was administered at the start of reperfusion [17]. Small interfering RNA (siRNA) targeted to Prdx6 (Prdx6-siRNA) and a scrambled RNA (scrRNA) controls were purchased from GenePharma Co., Ltd. (Shanghai, China). The sequence and antisequence were 5′-CUUCCACGAUUUCCUAGGATT-3′ and 5′-UCCUAGGAAAUCGUGGAAGTT-3′, respectively. 24 hours before middle cerebral artery occlusion (MCAO) surgery, 5 *μ*l of either Prdx6-siRNA or scrRNA was applied by intracerebroventricular injection at a rate of 0.2 *μ*l/min. The stereotactic coordinates were 1.5 mm lateral and 1.1 mm posterior to the bregma and 4.5 mm below the surface of the skull.

### 2.2. MCAO Model

MCAO was induced as previously described [11]. Briefly, all the rats were anesthetized with IP injections of 10% chloral hydrate (350 mg/kg). The right common carotid artery (CCA), the right external carotid artery (ECA), and the right internal carotid artery (ICA) were exposed through a short incision. ICA was clamped by using bulldog clamp temporarily. Then, monofilament nylon sutures with heat-blunted tip were placed into the ECA and advanced into the ICA approximately 18–20 mm. The suture was allowed to remain in the position for 2 hours and subsequently removed to allow reperfusion. To confirm the successful occlusion of the middle cerebral artery (MCA), cerebral blood flow (CBF) was monitored by laser Doppler flowmetry (Perimed, North Royalton, OH, USA) during occlusion and early reperfusion [18]. The standard I/R model was defined as a reduction of cortical CBF to 20–30% of baseline in the initial 30 minutes after occlusion and a 70% blood flow recovery during the first 10 minutes of reperfusion. The rectal temperature of rat was monitored and maintained at 37 ± 1°C with a head heating lamp during the surgical procedures. Sham groups received the same procedure without MCA occlusion. After withdrawing the suture and recovery from anesthesia, all rats were returned to their cages with free access to food and water.

### 2.3. Experimental Design for Rats Studies

*Protocol 1.* To determine the expression Prdx6 mRNA and protein after cerebral I/R and to identify the appropriate time point for experiments, the rats were randomly divided into six groups: sham group, I/R 6-hour group, I/R 12-hour group, I/R 24-hour group, and I/R 48-hour group (*n* = 8 for each group). All samples for this protocol were collected at 6 hours, 12 hours, 24 hours, and 48 hours after reperfusion. To determine the effects of Cur treatment on Prdx6 expression after cerebral I/R, rats were randomly divided into six groups: sham group, I/R group, Cur group, Cur + I/R group, I/R + siRNA group, and I/R + scrRNA group (*n* = 8 for each group). The Prdx6 mRNA and protein levels were measured 24 hours after reperfusion.

*Protocol 2.* To investigate the effects of Prdx6-konckdown on Cur-induced neuroprotection during I/R, Prdx6 siRNA was administered to I/R rats after Cur treatment. Rats were randomly divided into five groups: sham group, I/R group, Cur + I/R group, Cur + I/R + siRNA group, and Cur + I/R + scrRNA group (*n* = 8 for each group). Neurological score and infarct volumes were assessed. Levels of ROS, MDA, 8-hydroxy-2-deoxyguanosine (8-OHdG), and nitrotyrosine were measured for oxidative stress index measurements.

*Protocol 3.* To evaluate the regulatory effect of Cur treatment on Prdx6 expression via SP1, the rats were randomly divided into four groups: I/R group, Cur + I/R group, I/R + MTM group, and Cur + I/R + MTM group (*n* = 8 for each group). The expressions of Prdx6 mRNA and protein were assessed.

### 2.4. Neurobehavioral Evaluation and Infarct Volume Measurement

24 hours after reperfusion, a five-point scale with modifications was used to test neurological deficiency [16]. Neurological deficits were scored as follows: 0, no deficit; 1, fail to flex left forepaw fully; 2, decreased grip strength of left forepaw; 3, circling to left by pulling the tail; 4, spontaneous circling.

The brains were removed from rats, transferred immediately to −20°C for 20 minutes and then cut into 2 mm thick slices. The slices were stained with standard 2% TTC for 20 minutes at 37°C and photographed with a digital camera. Unstained areas were defined as infarct and were measured using image analysis software. The evaluation of infarct volume was performed using the following equation: relative infarct volume = (contralateral area − ipsilateral noninfarct area)/contralateral area [19].

### 2.5. Immunofluorescent Staining

Cryopreserved frozen brain tissues were prepared by cutting 10 *μ*m thick coronal sections that were 1.5 mm rostral to the bregma using a cryostat. Prdx6 labeling was performed by incubating sections with anti-Prdx6 rabbit polyclonal antibody (Abcam American; 1 : 100 dilution) and mouse anti-Neuronal Nuclei monoclonal antibody (NeuN, 1 : 500; Millipore) overnight at 4°C. After washing in phosphate-buffered saline (PBS), sections were incubated with green-fluorescent Alexa Fluor 488 and red-fluorescent Alexa Fluor 594 (1 : 500 for both; Vector Laboratories, Inc., Burlingame, CA) for 1 hour at room temperature. All sections were observed and the images were captured using a fluorescence microscope (BX51, Olympus, Tokyo, Japan).

### 2.6. Oxidative Stress-Related Biochemical Index Measurement

The peri-infarct cortex was separated and homogenized in ice-cold saline with a 1 : 9 weight-to-volume value. The homogenized samples were centrifuged at 2500 rpm for 15 minutes and supernatants collected. Oxidative stress was measured using markers, including ROS (Westang, Shanghai, China), MDA (Jiancheng, Nanjing, China), 8-OHdG (Cayman, USA), and nitrotyrosine (Millipore, USA), according to their kit instructions.

### 2.7. Western Blot

The peri-infarct cortex was homogenized in ice-cold RIPA lysis buffer (Beyotime, Haimen, China), which was added to 1% phenylmethanesulfonyl fluoride (PMSF). Total protein concentration was determined using bicinchoninic acid (BCA) protein assay kit (Beyotime, Haimen, China) according to the manufacturer's instructions. Equal amounts of protein were loaded onto 10% polyacrylamide gels, subjected to SDS-PAGE, and then transferred onto PVDF membranes (Millipore). The membranes were blocked with 5% nonfat dried milk for 2 hours at room temperature and incubated overnight at 4°C with anti-Prdx6 rabbit polyclonal antibody (Abcam, American; 1 : 1000 dilution). After washing, the membrane was incubated with secondary antibody for 1 hour at room temperature. Quantitative Western blot analysis was performed using enhanced chemiluminescent reagent (ECL; Millipore). The changes in protein activation are shown normalized to *β*-tubulin.

### 2.8. Real-Time PCR

TRIzol reagent (Takara, Japan) was used to extract total RNA from the cortex of the ischemia penumbra according to the manufacturer's instructions. Real-Time PCR protocol was 94°C for 30 seconds followed by 42 cycles of 5 seconds at 95°C and 30 seconds at 60°C. The primer sequences were designed as follows: Prdx6, forward primer 5′-CAACTTTGAGGCCAATACCA-3′, Prdx6, reverse primer 5′-CAACTTAACATTCCTCTTGG-3′, *β*-Tubulin, forward primer 5′-TCTTCCACCATGAGCAGCTC-3′, and *β*-Tubulin, reverse primer 5′-AACCTTGGAGACCAGTGCAG-3′.

### 2.9. Statistical Analysis

The neurologic scores were described as median ± interquartile range and analyzed by Kruskal-Wallis test followed with Mann–Whitney *U* test. Other data are expressed as mean ± standard deviation (SD). Differences between groups were analyzed by one-way analysis of variance (ANOVA) followed by Tukey's multiple comparison post hoc test. *p* < 0.05 was defined as statistically significant. The software SPSS 11.0 for Windows was used for statistical analysis.

## 3. Results

### 3.1. Curcumin Treatment Upregulated Neuronal Expression of Prdx6

**Figure 1:**
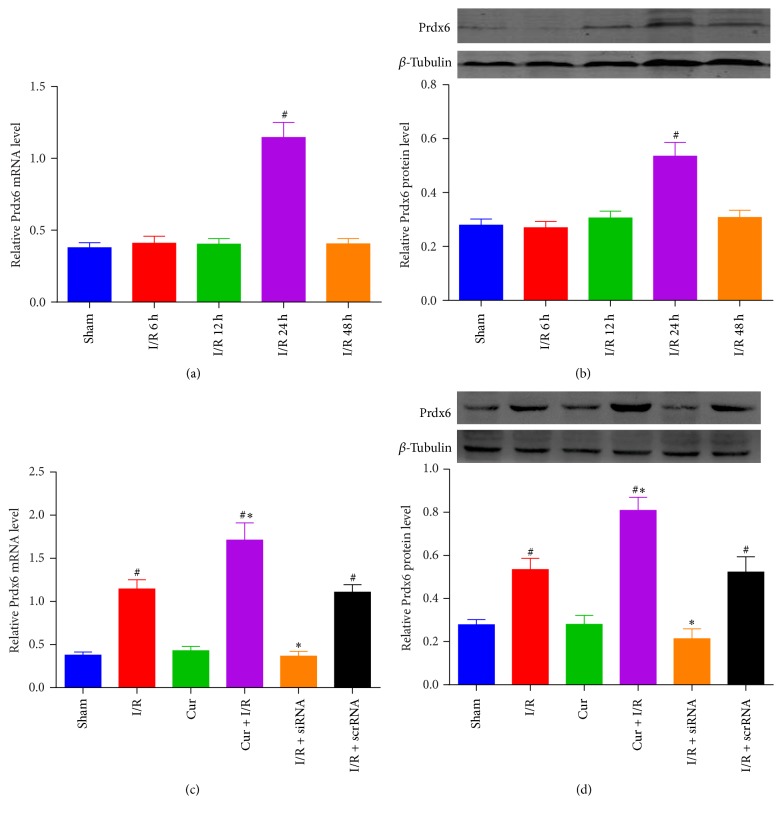
*The expression of Prdx6 in the peri-infarct cortex of rats after cerebral I/R injury*. (a) Real-Time PCR results show that Prdx6 mRNA levels are significantly increased 24 hours after I/R. (b) Western bolt analysis shows that Prdx6 protein levels demonstrate a significant increase after 24 h I/R. (c) Real-Time PCR results show that Prdx6 mRNA levels are inhibited by siRNA and increased by curcumin treatment. (d) Western bolt analysis shows that Prdx6 was inhibited by siRNA and increased by curcumin treatment. *n* = 4. ^#^*p* < 0.05 versus sham group. ^*∗*^*p* < 0.05 versus I/R group.

To assess the effects of Prdx6 on cerebral I/R injury, Prdx6 mRNA and protein expression in the peri-infarct cortex was investigated by RT-PCR (Figure 1(a)) and Western blot (Figure 1(b)) at 6, 12, 24, and 48 hours after reperfusion. Prdx6 mRNA and protein expression in the peri-infarct cortex increased at 24 hours after reperfusion, so we choose this time point for subsequent experiments. As described in Figures 1(c) and 1(d), Prdx6 mRNA and protein expression was increased by curcumin treatment compared with I/R only group, and was inhibited by siRNA.

### 3.2. Localization of Prdx6 in Ischemic Stroke Rats

**Figure 2:**
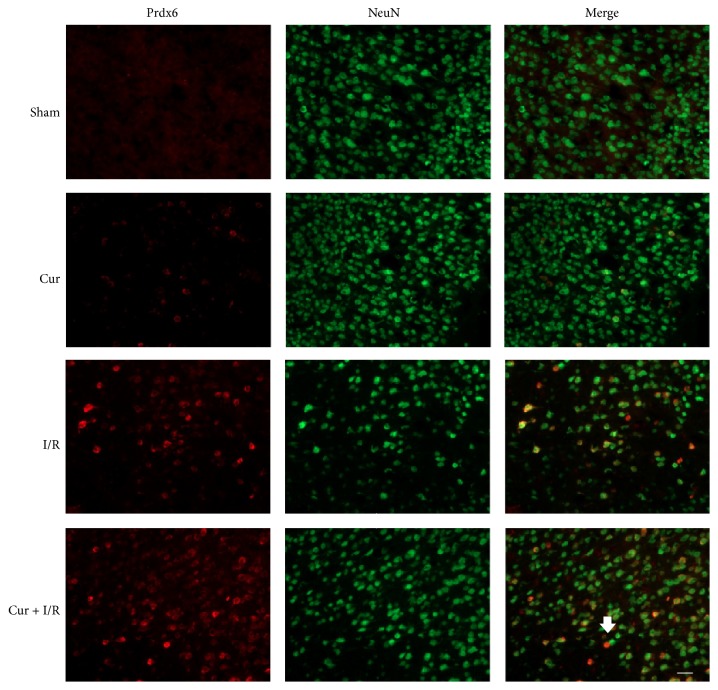
*Localization of Prdx6 in Ischemic Stroke Rats*. Immunofluorescent staining found that Prdx6 (red) was coexpressed with NeuN-positive neurons (green). *n* = 4. Scale bar = 20 *μ*m.

To determine the distribution of Prdx6, immunohistochemical staining was performed on the peri-infarct cortex. The result showed that Prdx6 was colocalized with NeuN, a neuronal marker (Figure 2).

### 3.3. Prdx6-siRNA Reversed the Neuroprotective Effects of Curcumin Treatment against Ischemic Injury

**Figure 3:**
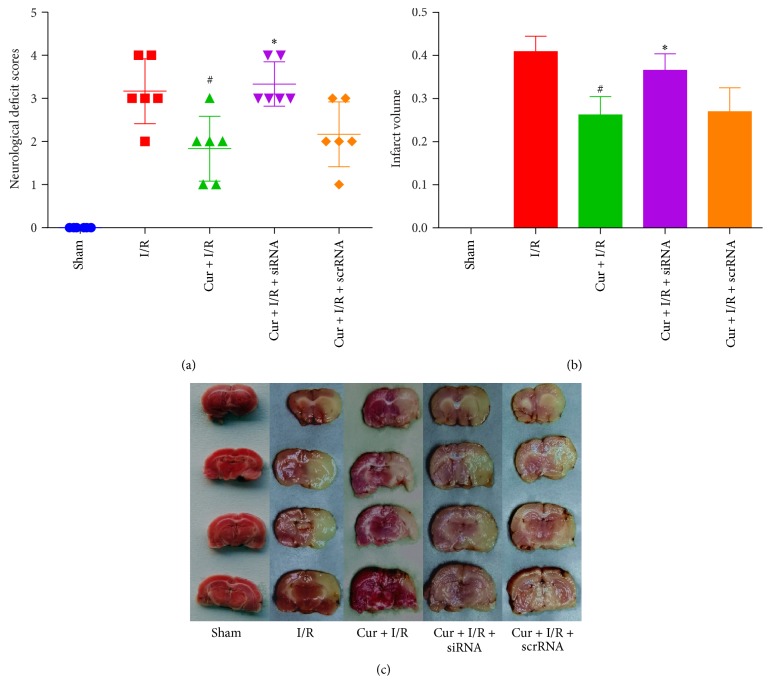
*Prdx6 knockdown reversed the curcumin-induced neuroprotective effects against cerebral I/R injury*. (a) Inhibition of Prdx6 reversed the curcumin-induced improvements on neurological scores and ((b) and (c)) infarct volume after reperfusion. *n* = 6. ^#^*p* < 0.05 versus I/R group. ^*∗*^*p* < 0.05 versus Cur + I/R group. Scale bar = 20 *μ*m.

As shown in Figure 3(a), Prdx6-siRNA reversed the improvement in neurological outcome induced by Cur treatment in the Cur + I/R + siRNA group versus Cur group. In Cur + I/R + scrRNA group, no statistically significant difference were observed in cerebral infarct size or neurological score compared to Cur + I/R group. In addition, as shown in Figures 3(b) and 3(c), the deficiency of Prdx6 weakens the cerebral infarct size by Cur treatment as compared to I/R group.

### 3.4. Prdx6-siRNA Weakens the Preventive Effect of Curcumin Treatment on Ischemic Oxidative Stress

**Figure 4:**
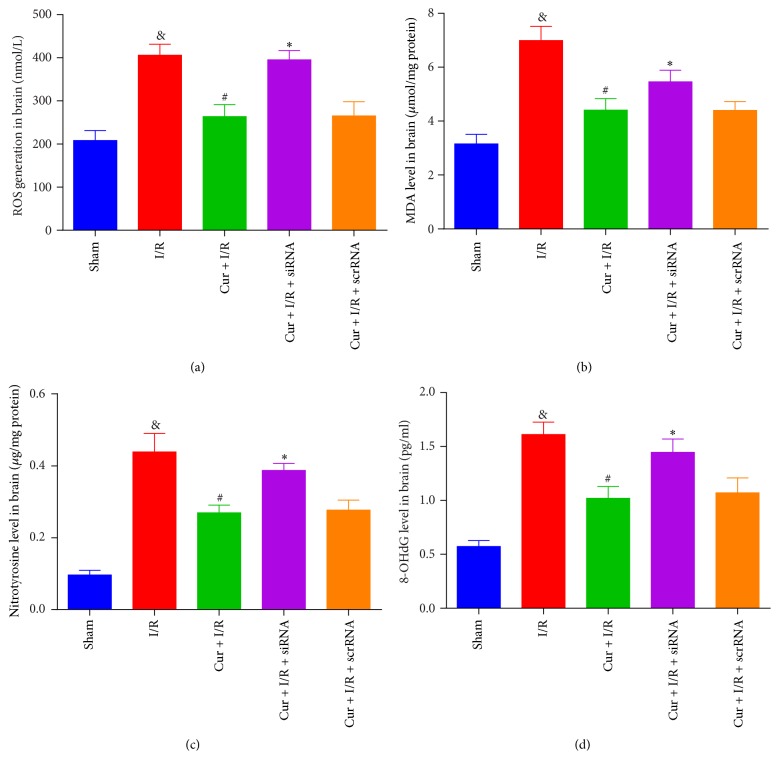
*Prdx6 knockdown abolished the preventive effect of Cur treatment against oxidative stress*. The levels of (a) ROS, (b) MDA, (c) nitrotyrosine, and (d) 8-OHdG were measured and compared to sham, I/R, Cur + I/R, Cur + I/R + siRNA, and Cur + I/R + scrRNA groups. *n* = 4. ^&^*p* < 0.05 versus sham group, ^#^*p* < 0.05 versus I/R group, and ^*∗*^*p* < 0.05 versus Cur + I/R group.

Oxidative stress was measured by ROS, MDA, nitrotyrosine, and 8-OHdG 24 hours after reperfusion (Figures 4(a), 4(b), 4(c), and 4(d)). Cur treatment significantly reduced the generation of ROS, MDA, 8-OHdG, and nitrotyrosine compared to the I/R only group. The levels of ROS, MDA, nitrotyrosine, and 8-OHdG in Cur + I/R + siRNA group were similar as those in I/R group. These data indicate that Prdx6 siRNA reversed the antioxidant effect of Cur treatment.

### 3.5. Upregulation of Prdx6 by Curcumin Treatment Was SP1-Dependent

**Figure 5:**
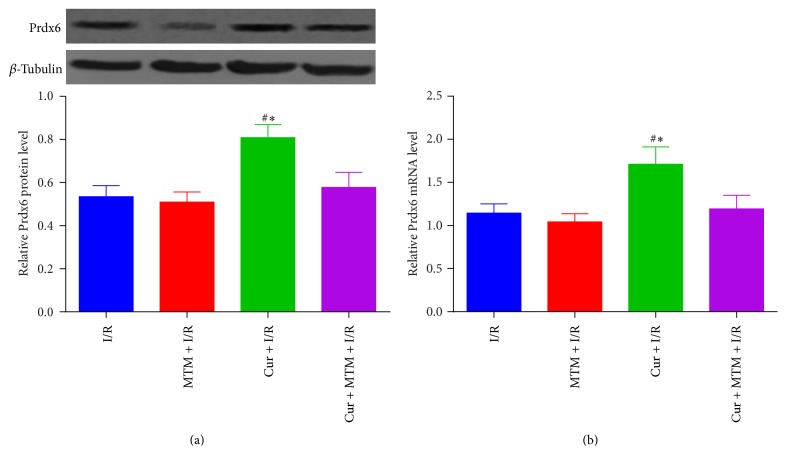
*Cur treatment increased Prdx6 mRNA and protein expression via SP1*. (a) Western bolt analysis demonstrated that Prdx6 protein levels significantly increased in Cur + I/R groups. (b) Real-Time PCR results show that Prdx6 mRNA levels significantly increased in Cur + I/R groups. *n* = 4. ^#^*p* < 0.05 versus I/R group and ^*∗*^*p* < 0.05 versus Cur + MTM + I/R group.

As presented in Figures 5(a) and 5(b), pretreatment with MAM, the SP1 antagonist, decreased the expression of Prdx6 mRNA and protein in Cur + I/R + MTM groups compared with Cur + I/R group.

## 4. Discussion

This study found that Cur treatment increased Prdx6 mRNA and protein expression, and attenuated neurological deficits, infarct volume, and oxidative stress after I/R. These beneficial effects were reversed by Prdx6-siRNA. Taken together, these results indicate that upregulation of Prdx6 is involved in Cur-mediated protection against transient focal cerebral I/R in vivo. Furthermore, treatment with the SP1 antagonist MTM abolished the Prdx6 upregulation induced by Cur treatment. These findings suggest that Prdx6 upregulation by Cur treatment is mediated through SP1, in rats after stroke. This is a novel mechanism of Cur-mediated protection against transient focal cerebral I/R in vivo.

Cur possessed a variety of pharmacological and biological properties, including neuropotetion [20, 21]. Cur upregulates Prdx6 in HT22 cells, human lens epithelial cells, astrocytes [13, 14, 22]. Prdx6 is an antioxidant expressed in the lungs, brain, liver, and kidneys [23–26]. Spermatozoa lacking PRDX6 showed significantly increased levels of oxidative damage as evidenced by high levels of lipid peroxidation, DNA oxidation, and protein oxidation [27]. Prdx6 provided protection against stresses by clearing the oxidative load in lens epithelial cells [28] and in neuronal cells [24]. In our study, the expression of Prdx6 mRNA and protein increased in the ischemic hemispheres after reperfusion and peaked at 24 hours. Prdx6 is expressed in numerous cells in the central nervous system under different conditions. Prdx6 is highly expressed in rat astrocytes after traumatic brain injury [29] and in A*β*_1–42_/H_2_O_2_-induced neuronal cells where it functions to protect against thiacremonone-induced oxidative stress [24]. Additionally, Prdx6 is expressed in neuronal cells in response to oxidative stress induced by 4-HBA treatment [10]. Here, we observed Prdx6 expression in neurons, consistent with previous studies [10, 24]. Our study went further to demonstrate that expression of Prdx6 mRNA and protein is increased in the Cur + I/R group relative to I/R group, suggesting that Cur treatment upregulates the expression of Prdx6 after ischemic stroke. SiRNA studies demonstrated the neuroprotective effects of Cur are mediated by Prdx6 during ischemic injury. Microinjection of Prdx6 siRNA reduced infarct volume and neurological deficit induced by Cur treatment following I/R. Taken together, these data strongly implicate Prdx6 in the neuroprotective effects of Cur treatment.

Oxidative stress, apoptosis, and inflammation participate in the pathogenesis of ischemic stroke [3]. Reactive oxygen species (ROS) are free radicals that damage DNA and RNA, oxidize proteins, lipids, and polysaccharides, and induce cell apoptosis or necrosis under conditions of oxidative stress [30–32]. Reactive nitrogen species (RNS) can cause lipid peroxidation [33]. 8-OHdG is a biomarker of DNA damage induced by oxidative stress [34], MDA is a biomarker of lipid peroxidation [35], and nitrotyrosine is a biomarker of RNS induced oxidant stress and lipid peroxidation [36]. Here, we examined the level of these biomarkers to assess the oxidative stress and demonstrated that Cur treatment reduced levels of these oxidative products, while Prdx6 siRNA reversed this antioxidant effect. Based on these results, the antioxidant effects of Cur are mediated by Prdx6. Prdx6 is a bifunctional protein with peroxidase activity and PLA2 activity. Peroxidase activity of Prdx6 facilitates the growth of lung cancer cells, and PLA2 activity promotes invasiveness [37]. We speculate that the antioxidant effects of Prdx6 following I/R are associated with the peroxidase activity of Prdx6 although further studies are needed to verify this hypothesis.

We have developed the following mechanism of neuroprotection by Cur treatment during I/R: Cur alleviates oxidative stress in the endoplasmic reticulum (ER) by increasing Prdx6 expression in HT22 cells [13]. Since the Prdx6 promoter contains three SP1 sites and all sites contribute to Prdx6 transcription [14], we propose Cur activation of SP1 induces upregulation of Prdx6 which functions as an antioxidant during I/R.

In conclusion, our data indicate that Prdx6 is involved in the suppression of oxidative stress induced by Cur during I/R. Downregulation of Prdx6 reversed the antioxidant effect induced by Cur in the cerebral tissue of I/R rats and Prdx6 activation was induced by Cur via SP1. This study reveals a novel antioxidant mechanism of Cur treatment mediated by SP1/Prdx6 pathway.
